# Integrin β1 Promotes Pancreatic Tumor Growth by Upregulating Kindlin-2 and TGF-β Receptor-2

**DOI:** 10.3390/ijms221910599

**Published:** 2021-09-30

**Authors:** Md Saimon Mia, Yagna Jarajapu, Reena Rao, Sijo Mathew

**Affiliations:** 1 Department of Pharmaceutical Sciences, School of Pharmacy, North Dakota State University, Fargo, ND 58108-6050, USA; mdsaimon.mia@ndsu.edu (M.S.M.); yagna.jarajapu@ndsu.edu (Y.J.); 2 Kidney Institute, University of Kansas Medical Center, Kansas City, KS 66160, USA; rrao@kumc.edu

**Keywords:** integrin β1, kindlin-2, TGF-β receptor, pancreatic cancer

## Abstract

The tumor microenvironment plays a critical role in defining the growth and malignancy of solid tumors. Extracellular matrix (ECM) proteins such as collagen, vitronectin, and fibronectin are major components of the tumor microenvironment. Tumor growth-promoting reciprocal interaction between ECM and cytoplasmic proteins is regulated by the cell surface receptors called integrins. This study investigated the mechanism by which integrin β1 promotes pancreatic tumor growth. In MIA PaCa-2 pancreatic cancer cell line, the loss of integrin β1 protein reduced the ability of cells to proliferate in a 3D matrix and compromised the ability to form a focal adhesion complex. Decreased expression of integrin α5 was observed in KO cells, which resulted in impaired cell spreading and adhesion on vitronectin and fibronectin. Reduced expression of the integrin-associated protein, kindlin-2 was also recorded. The downregulation of kindlin-2 decreased the phosphorylation of Smad2/3 by reducing the expression of TGF-β receptor 2. These results unravel a new mechanism of integrin β1 in tumor growth by modifying the expression of kindlin-2 and TGF-β receptor 2 signaling.

## 1. Introduction

Increased ECM deposition in the tumor microenvironment is a characteristic of solid tumors, which promotes disease progression, interferes with patient prognosis, and is an obstacle for chemotherapeutic interventions. Pancreatic cancer has a high mortality rate with 92% of patients dying within five years of diagnosis, and in 2020, approximately 57,600 people were diagnosed with pancreatic cancer in the USA [1]. A clear understanding of the cellular signaling associated with stromal fibrosis in pancreatic cancer is critical for developing therapeutic strategies for pancreatic cancer to circumvent the shortcomings in the current therapeutic regimen. Integrins are a family of transmembrane adhesion receptors which transduce the structural and compositional alterations of tumor stroma to cellular functions. Integrin expression is higher in pancreatic tumor cells compared to normal tissues [2,3,4]. The mechanisms by which integrin promotes tumor growth in pancreatic cancer are unclear. Generally, integrin receptors exist as non-covalently bound heterodimers of α and β subunits and are implicated in various stages of cancer progression. Interaction of integrin with different cytosolic adaptor proteins is known to activate or inhibit various cellular signaling pathways. Kindlin-2 is one of these adaptor proteins that interacts with integrin and facilitates integrin activation and outside-in cellular signaling in normal epithelial cells [5,6,7]. Kindlin-2 also regulates actin dynamics and focal adhesions [8,9,10]. In pancreatic tumors, kindlin-2 is a prognostic marker as the mRNA level is higher in tumor tissues compared to normal pancreatic tissues [11,12,13]. Kindlin-2 is also important in promoting multiple cellular signaling pathways promoting metastasis in pancreatic cancer.

TGF-β is an important tumor-promoting cytokine, abundant in the tumor microenvironment, which mostly exists in an inactive conformation. Latent TGF-β is inactive in the extracellular matrix and exists as a complex with the latency-associated peptide (LAP). Conformational changes induced by the binding of latent TGF-β LAP domain with integrin β6 and β8 is critical for the activation of TGF-β [14,15]. The TGF-β superfamily of cytokines transduces cell signaling through the TGF-β receptor-2 and TGF-β receptor-1 complex. After the binding of active TGF-β1, TGF-β receptor-2 trans-phosphorylates TGF-β receptor-1 leading to the phosphorylation of Smad transcription factors. In normal cells and early carcinomas, TGF-β exerts tumor suppressor functions by a protective anti-proliferative effect and these cytostatic effects are often lost in advanced tumors. In solid tumors, increased activation of TGF-β induces actin polymerization, cytoskeletal remodeling, and the formation of actin stress fibers leading to the deposition of the fibrotic stroma. A feedback loop between kindlin-2 and TGF-β in breast, esophageal and pancreatic cancer has also been reported [16]. Components of the molecular link, as well as the reciprocal functional regulation between the intracellular TGF-β mediated signaling pathways and stromal proteins in the tumor microenvironment are not known.

This study evaluated the role of integrin β1 in the reciprocal regulation between stromal proteins and TGF-β signaling. Genetic deletion of integrin β1 decreased tumor growth of pancreatic cancer cell line in 3D gels. Integrin β1 gene deletion also reduced cell adhesion and spreading on fibronectin and vitronectin by decreasing integrin α5 expression. Results also showed that integrin β1 depletion attenuates focal adhesion complex formation by downregulating kindlin-2 and TGFβ receptor 2 expression leading to reduced Smad2/3 phosphorylation.

## 2. Results

### 2.1. Integrin β1 Promotes Pancreatic Tumor Growth

**Figure 1:**
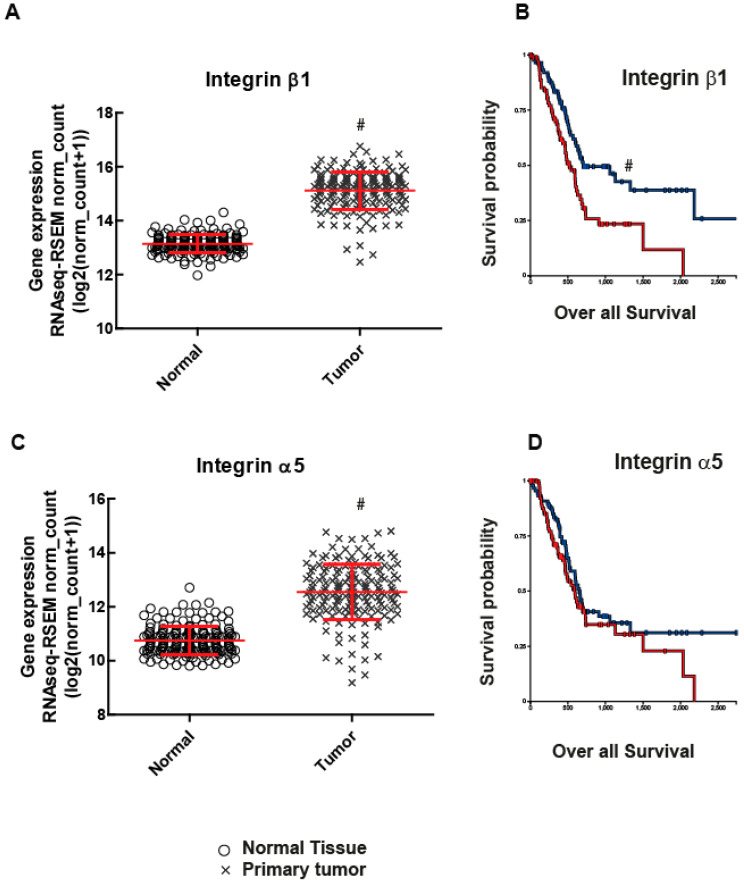
Integrin α5 and β1 are associated with pancreatic cancer patient survival. Integrin β1 and α5 mRNA levels in primary tumors were compared with normal tissue using TCGA data. (**A**) Primary tumors have higher gene expression of integrin β1 than normal tissues (# *p* < 0.0001, *n* = 165 for normal and *n* = 178 for tumors) red lines indicate the mean and SD of the values. (**B**) Increased expression of integrin β1 (red lines) associates with lower survival in patients as depicted by KM plot *p* < 0.001. (**C**) Integrin α5 also has higher gene expression than normal tissues (# *p* < 0.0001, *n* = 165 for normal and *n* = 183 for tumors). (**D**) Integrin α5 did not show a clear relationship with patient survival *p* > 0.05.

**Figure 2:**
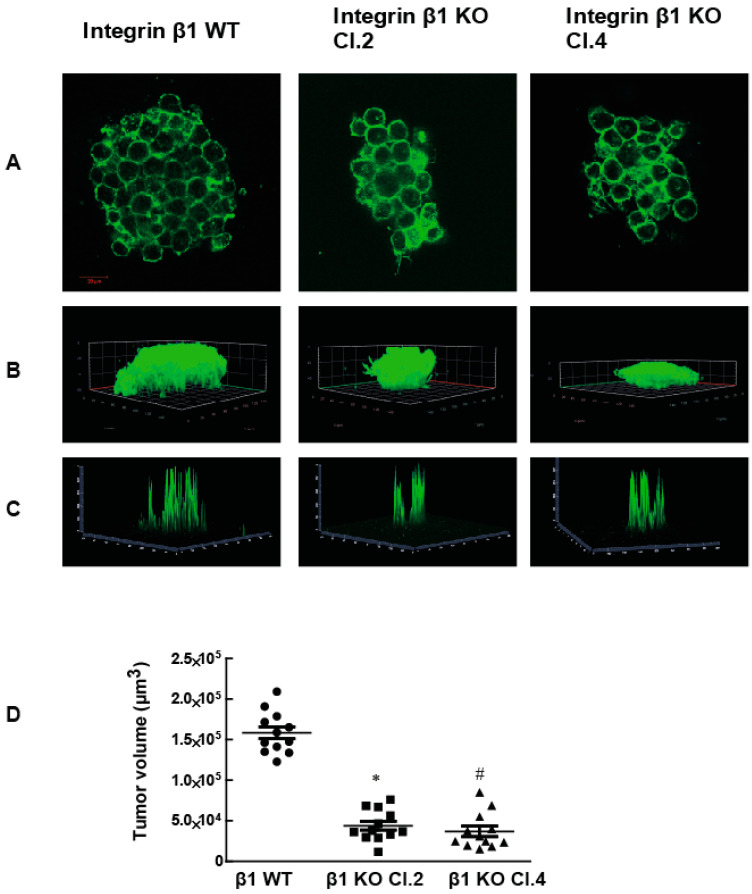
The deletion of the β1 integrin gene from MIA PaCa-2 cells decreases tumor growth on the 3D matrix. (**A**) Tumors formed by MIA PaCa-2 cells expressing the integrin β1 gene and cells where the integrin β1 gene removed were stained for F-Actin (phalloidin, green) to estimate the size of the tumor. Scale bar 20 µm. (**B**) 3D projection of z stack indicate the tumor size. (**C**) The projection of the intensity of actin staining (view 2.5D) and **(D)** Tumor volumes were measured after the acquisition of z-stack slices with confocal microscope. These images were processed using ImageJ software to determine the volume. One-way ANOVA was performed with Bonferroni’s test to compare between groups. * indicates a statistically significant difference between WT and Clone 2, *n* = 12, *p* < 0.001). # indicates the statistically significant difference between groups WT and Clone 4 *n* = 12, *p* < 0.001. Data shown are mean ± SEM, unit: µm^3^.

**Figure 3:**
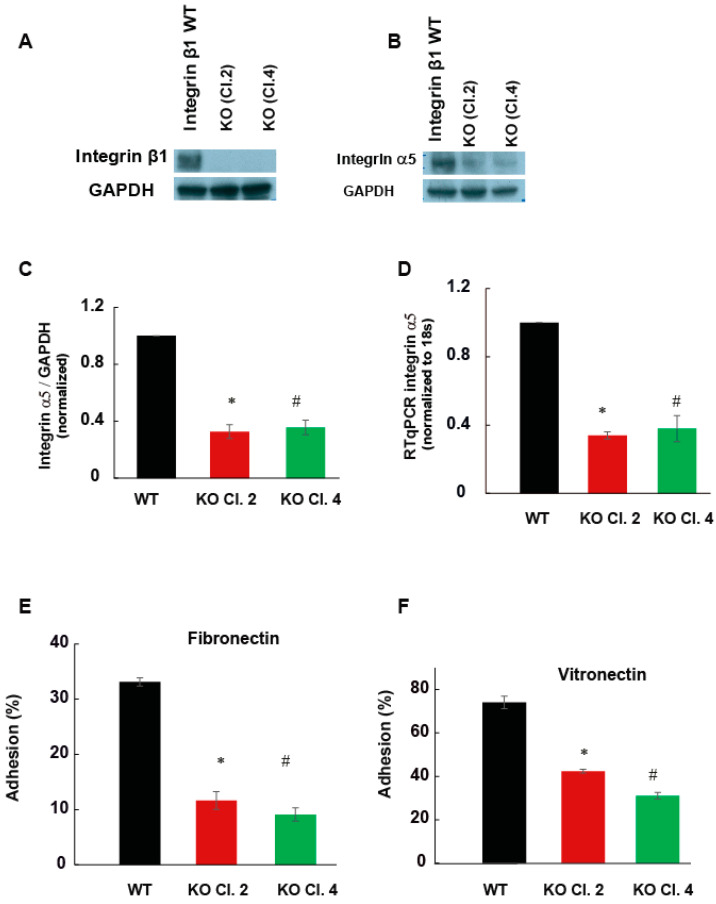
Integrin β1 deletion decreased the cellular adhesion of MIA PaCa-2 cells to vitronectin and fibronectin. (**A**) Integrin β1 expressing MIA PaCa-2 and integrin β1 KO clones were immunoblotted by anti-integrin β1 antibody. GAPDH was used as the loading control. (**B**) Cell lysate was immunoblotted with integrin α5 antibodies. GAPDH was used as the loading control. (C) Quantification of integrin α5 expression normalized to GAPDH, mean ± SEM from three independent experiments (*n* = 3). One-way ANOVA performed with Bonferroni’s test indicated a statistically significant decrease in cell adhesion, * *p* < 0.001 between WT and clone2, # *p* < 0.001 between WT and clone4. (**D**) The expression of integrin α5 mRNA by RT-PCR. ΔΔCt values normalized with to 18s *n* = 3, * *p* < 0.001 between WT and clone2, # *p* < 0.001 between WT and clone4. (**E**) Percentage change in cellular adherence of integrin β1 KO to fibronectin (5 µg/mL) compared to the integrin β1 expressing cells, *n* = 3, * *p* < 0.01 between WT and clone2, # *p* < 0.01 between WT and clone4. (**F**) Comparison of cellular adhesion between integrin β1 expressing MIA PaCa-2 cells and integrin β1 KO cells to vitronectin (5 µg/mL) *n* = 3, * *p* < 0.01 between WT and clone2, # *p* < 0.01 between WT and clone4.

To examine the role of integrin β1 in pancreatic cancer, we first examined RNA sequence data from the TCGA database. Integrin β1 expression was found to be significantly higher in primary pancreatic human patient tumor samples when compared to normal pancreatic tissue (Figure 1A *p* <0.0001). Moreover, Kaplan Meier Curve survival plot analysis revealed that the integrin β1 expression in pancreatic tumors negatively correlated with patient survival *p* <0.001) (Figure 1B). Although there is an increase in the expression of Integrin α5 in human pancreatic tumor patients compared to normal pancreatic tissues (*p* < 0.0001), it does not show any clear relationship with patient survival (*p* > 0.05) (Figure 1C,D). To examine the mechanism for integrin β1 mediated pathogenicity in pancreatic ductal adenocarcinoma, we used MIA PaCa-2 cell lines, a human pancreatic cancer cell model [17]. We generated integrin β1 gene deleted MIA PaCa-2 cells using CRISPR-Cas9 technology (Santa Cruz Biotechnology, Dallas, TX, USA) and selected two clones (integrin β1 KO clone 2 and clone 4) by puromycin selection (Figure 3A and Figure S1). Integrin β1 gene deleted cells were embedded in 3D matrigel and grown for 7 days and the tumors were imaged using confocal microscopy (Zeiss LSM 900, Carl Zeiss Microscopy, LLC, Thornwood, NY, USA) after staining with actin. While MIA PaCa-2 cells expressing wild type (WT) integrin β1 proliferated and formed tumor like colonies inside 3D gel (Figure 2A). The integrin β1 KO clones significantly reduced cell growth and formed smaller tumor like colonies (Figure 2A,B). 3D projection and intensity projection (2.5D) showed that tumor volumes were 70% reduced in the integrin β1 KO clones compared to integrin β1 WT cells (Figure 2C,D).These data suggest that integrin β1 inhibition could reduce pancreatic cancer cell proliferation and tumor growth. Similar observations reported in an earlier study using mice pancreatic tumor cell lines iKras*p53* also support our finding in MIA PaCa-2 cells [18].

### 2.2. Adhesion of MIA PaCa-2 Cells to Vitronectin and Fibronectin Is Decreased by the Deletion of Integrin β1

Integrin is a heterodimeric receptor composed of α and β subunits, which bind to extracellular matrix proteins. The effect of the protein deletion was confirmed by Western blotting (Figure 3A). To understand the specific integrin heterodimer involving integrin β1 in pancreatic tumor growth, functional assays were performed using selective extracellular matrix proteins. The expression of integrin α5 decreased significantly by approximately 75% in the integrin β1 KO MIAPaCa-2 cells compared to integrin β1 WT MIA PaCa-2 cells, shown by mRNA and protein levels (Figure 3B–D). α5β1 is known to mediate the adhesion of pancreatic cells to fibronectin and vitronectin.

To validate the functional significance of decreased integrin α5, cell adhesion assays were performed on matrix proteins fibronectin and vitronectin. Integrin β1 expressing MIA PaCa-2 cells and integrin β1 KO clones were allowed to adhere on matrix coated plates for 1 h at 37 °C. After removing the non-adherent cells, the number of cells adhered to the matrix was determined using Janus green staining. Integrin β1 deletion significantly decreased cell adhesion of MIA PaCa-2 cells to these matrix proteins (*p* < 0.01). In integrin β1KO clones, the cell’s adhesion to fibronectin and vitronectin decreased by 50% compared to integrin β1 expressing cells (Figure 3E,F). Previously, the fast-growing metastatic variant of the pancreatic cancer cell line Colo-357 showed decreased adhesion to collagen, vitronectin and laminin by the deletion of integrin β1 (3). Our data suggests that MIA PaCa-2 cells mainly promote tumor growth through integrin α5β1.

### 2.3. Integrin β1 Deletion Impaired the Cell Spreading and Focal Adhesions of MIA PaCa-2 Cells

**Figure 4:**
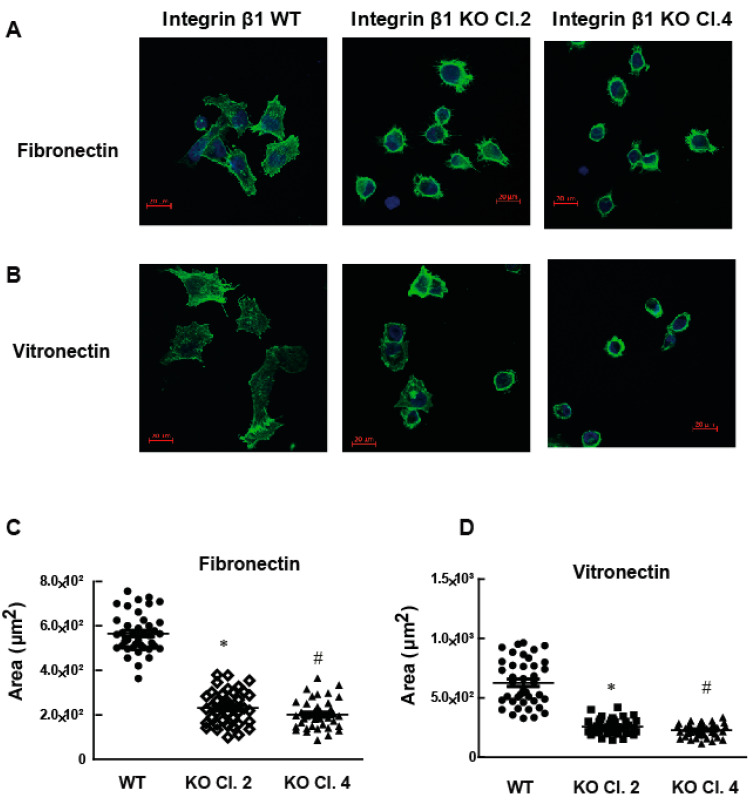
MIA PaCa-2 integrin β1 KO cells have decreased spreading ability compared to integrin β1 expressing cells. Cells were plated on either fibronectin or vitronectin coated plates for 4 h and the cells were stained with actin. (**A**) Actin staining of cell spread on 5 µg/mL fibronectin visualized by immunostaining (F-actin, phalloidin, green; and nucleus, DAPI, blue). Scale bar 20 µm. (**B**) Actin staining of cell spread on vitronectin visualized by immunostaining (Green: F-actin using phalloidin, and Blue: nucleus DAPI). Scale bar 20 µm. (**C**,**D**) Quantitative assessment of cell spreading in fibronectin (area: µm^2^). One-way ANOVA was performed with Bonferroni’s post-hoc test to compare between groups. * Indicates a statistically significant difference between groups (integrin β1 expressing MIA PaCa-2 and integrin β1 KO Clone 2) (*n* = 40, *p* < 0.001). # Shows a statistically significant difference between groups (integrin β1 expressing MIA PaCa-2 and integrin β1 KO Clone 4) (*n* = 40, *p* < 0.001). Data shown are mean ± SEM, unit: µm^2^.

**Figure 5:**
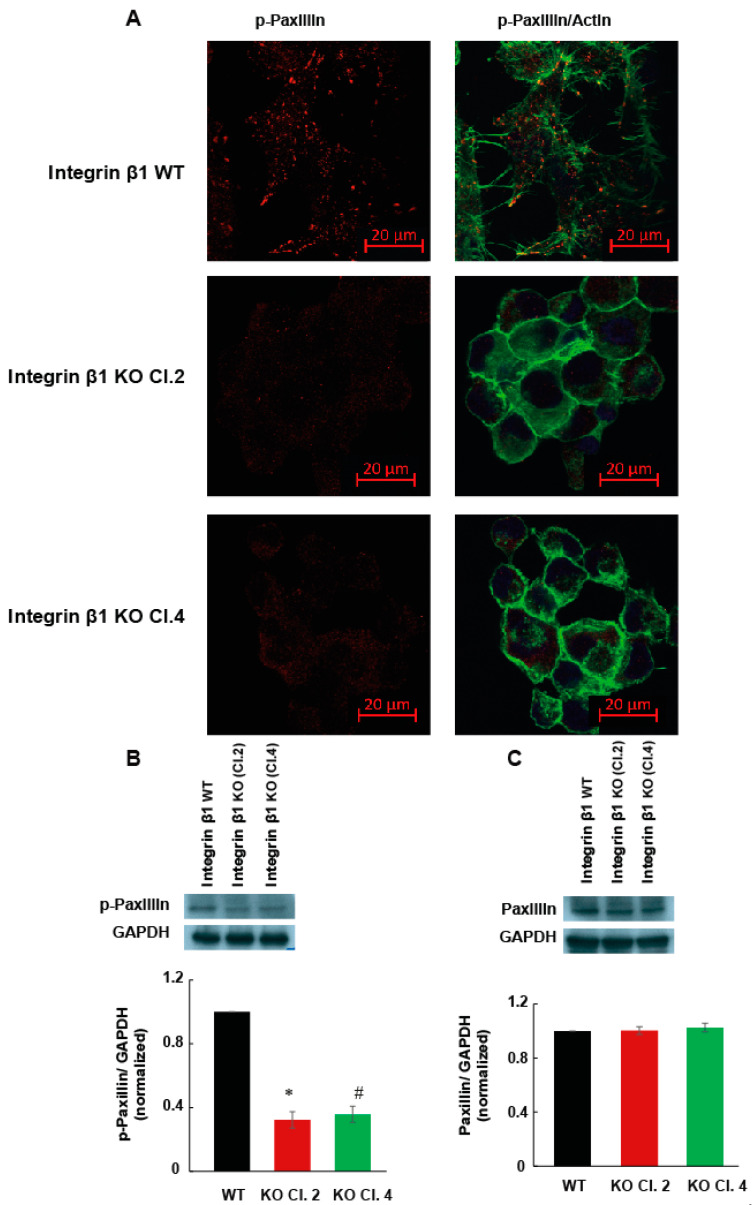
Impaired focal adhesion contributes to less cell spreading in MIA PaCa-2 integrin β1 KO cells (**A**) Phospho-paxillin immunostaining shows the decrease in the phosphorylation status of paxillin in MIA PaCa-2 and integrin β1 KO cells. Visualized by immunostaining F-actin, phalloidin, green, p-paxillin, Alexa flour 555, red). Scale bar 20 µm. (**B**) Immunoblotting confirmed the downregulation of p-paxillin. Normalized p-paxillin expression with GAPDH shows a significant decrease in expression of p-paxillin. (**C**) No significant change in paxillin expression normalized with GAPDH between WT and clones. One-way ANOVA was performed by Bonferroni’s test to compare between groups (*n* = 3). * indicates a statistically significant difference between groups (integrin β1 expressing MIA PaCa-2 and integrin β1 KO cells Clone 2, *p* < 0.001). # Shows a statistically significant difference between groups (integrin β1 expressing MIA PaCa-2 and integrin β1 KO cells Clone 4, *p* < 0.001).

Another important cell function associated with integrin heterodimer is the ability of cells to spread on the matrix proteins. Cell spreading is important for the formation of actin stress fibers and cytoskeleton mediated cell functions that induce the metastasis. Increase in the amount of matrix protein due to fibrosis induces matrix stiffness and cell spreading. To determine the role of integrin β1 on cell spreading, MIA PaCa-2 cells expressing integrin β1 and integrin β1 KO clones were allowed to spread on fibronectin or vitronectin coated plates for 4 h at 37 °C. Actin stress fibers were visualized by phalloidin staining and confocal microscopy. MIA PaCa-2 cells expressing integrin β1 did not show any significant change in spreading ability between fibronectin and vitronectin (Figure 4A,B). Cells expressing integrin β1 spread and formed well-defined actin filaments on these matrix proteins. The decrease in the extent of spreading was significant (*p* < 0.001) in both selected integrin β1 KO clones compared to cells expressing integrin β1 proteins (Figure 4C,D). Spreading and attachment of cells to ECM depend on the ability of cells to form focal adhesions. Focal adhesions are the major signaling hub that facilitate the reciprocal functional regulation between cytoskeleton and ECM. The phosphorylation status of paxillin is a direct measure of focal adhesion complexes and was measured by immunofluorescence and WB (Figure 5A,B). Phosphorylation of paxillin decreased significantly in integrin β1 KO cells. Total expression of paxillin did not change by the deletion of integrin β1 (Figure 5C).

### 2.4. Integrin β1 Regulates the Expression of Kindlin-2

**Figure 6:**
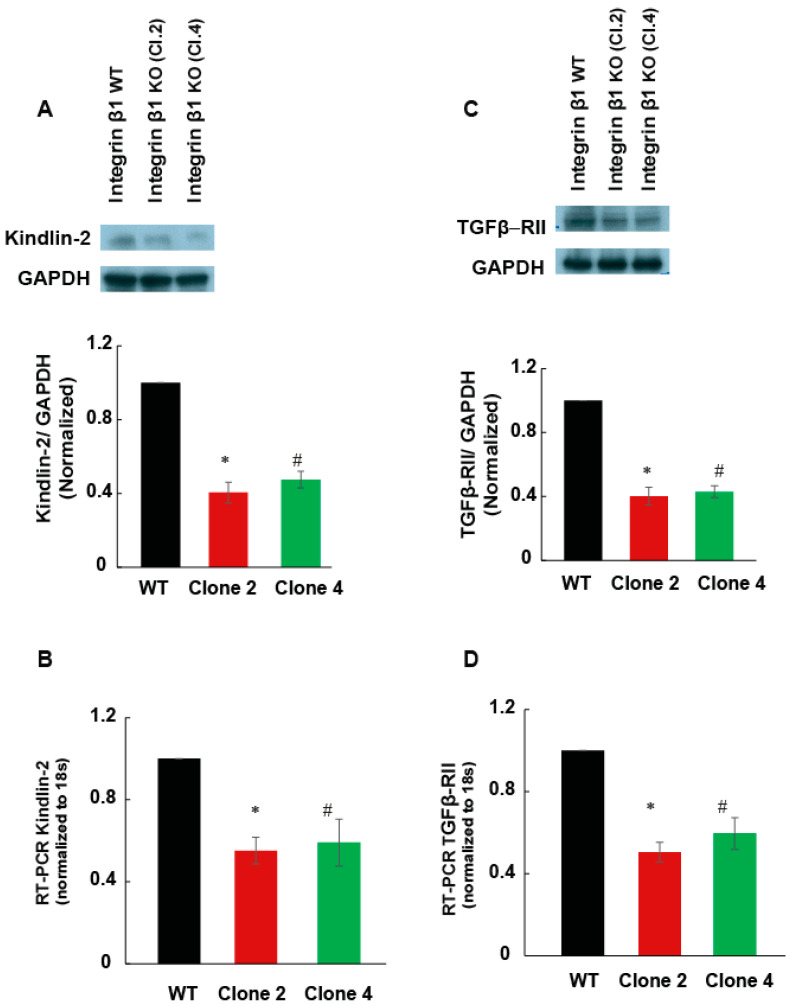
Integrin β1 deletion downregulated Kindlin-2 and TGFβ receptor 2 expression. (**A**) Cell lysate (30 µg/lane) from integrin β1 expressing MIA PaCa-2 and integrin β1 KO clones are immunoblotted for kindlin-2. Normalized protein expression with GAPDH shows a decrease in kindlin-2 expression in clones compared to the normal MIA PaCa-2 cells. (**B**) The mRNA expression quantified by RT-PCR analysis shows a significant decrease in the expression of kindlin-2 by the deletion of integrin β1. (**C**) Lysates were immune blotted with antibody recognizing TGFβ receptor 2. GAPDH is used as the loading control. Normalized protein expression shows downregulation of TGFβ receptor 2. (**D**) The mRNA expression quantified by RT-PCR analysis shows a significant decrease in the expression of TGFβ receptor 2 by the deletion of integrin β1. One-way ANOVA was performed by Bonferroni’s test to compare integrin β1 expressing MIA PaCa-2 and integrin β1 KO clones indicate a statistically significant difference *n* = 3, * WT and Clone 2 *p* < 0.001; # WT and Clone 4 *p* < 0.001. Quantification is expressed as mean ± SEM.

Kindlin-2 is a cytosolic adaptor protein associated with integrin β1 and is important for integrin clustering and signaling. One of the steps that leads to the formation of focal adhesions includes the complex formation between integrin β1 and kindlin-2. In addition, kindlin-2 is important in recruiting another major component of focal adhesions, paxillin, to this multi-protein molecular complex. Since we observed a decrease in the phosphorylation of paxillin (Figure 5A,C), the expression of kindlin-2 was investigated to see whether the kindlin-2 is essential for integrin β1 mediated focal adhesion formation. Kindlin-2 expression decreased significantly in integrin β1 KO cells compared to integrin β1 expressing MIA PaCa-2 cells (Figure 6A, *p* < 0.001). This was further confirmed by RT-PCR (Figure 6B, *p* < 0.001). Approximately 50% reduction in kindlin-2 expression was observed in integrin β1 KO cells compared to integrin β1 expressing MIA PaCa-2 cells. These data suggest that the decreased focal adhesion complex formation in integrin β1 KO cells is largely due to the decreased expression of Kindlin-2.

### 2.5. Integrin β1 Regulates the Expression of TGF-β Receptor 2 and TGF-β Cell Signaling in MIA PaCa-2 Cells

**Figure 7:**
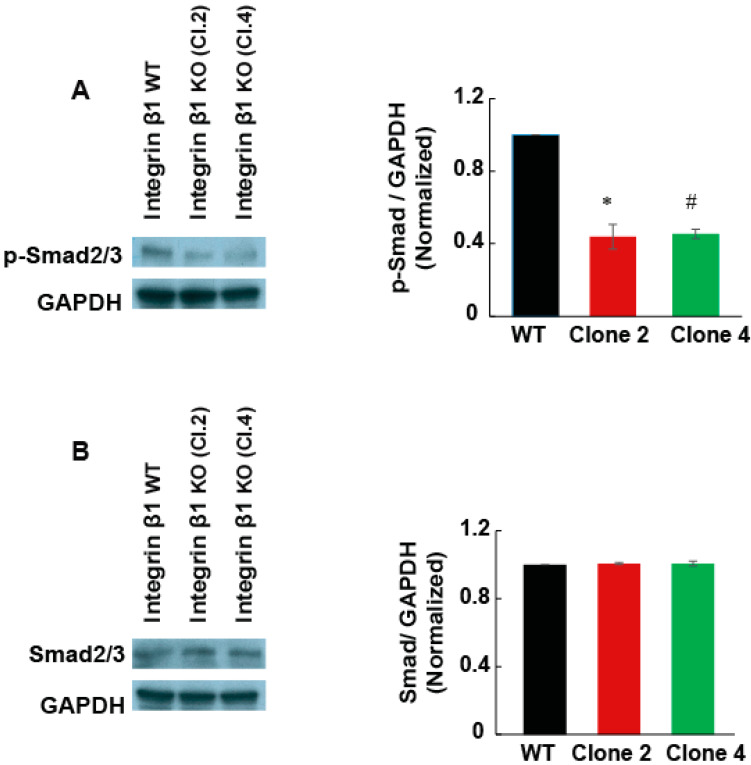
Integrin β1 deletion downregulated Smad2/3 phosphorylation. (**A**) Cell lysate from integrin β1 expressing MIA PaCa-2 and integrin β1 KO clones is immunoblotted for p-Smad2/3. (**A**) Immunoblotting of p-Smad2/3 shows inhibited downstream cell signaling pathways. Normalized P Smad-2/3 is shown using GAPDH as control. (**B**) Total expression of Smad-2/3 shows no significant change in the expression of Smad-2/3. One-way ANOVA performed with Bonferroni’s test to compare integrin β1 expressing MIA PaCa-2 and integrin β1 KO clones indicates a statistically significant difference *n* = 3, * WT and Clone 2 *p* < 0.001; # WT and Clone 4 *p* < 0.001. Quantification is expressed as mean ± SEM.

Kindlin-2 plays an important role in TGF-β mediated cell signaling in breast cancer cells, chondrocytes, and renal fibroblasts [19,20,21]. Since TGF-β1 signaling promotes fibrosis in pancreatic tumor, we examined if integrin β1 regulates TGF-β1 receptor expression or intracellular cell signaling. We found that the TGF-β receptor 2 mRNA and protein expression were significantly reduced (50%, *p* < 0.001), in integrin β1 KO cells, compared to integrin β1 WT cells (Figure 6C,D). Integrin β1 KO cells also showed a significant reduction in Smad2/3 phosphorylation indicating reduced TGF-β1-mediated intracellular cell signaling (Figure 7A,B).

## 3. Discussion

Integrins have been implicated in many tumor-promoting cell functions and are an effective target for cancer treatment [18,22,23]. However, many small molecule inhibitors or antibodies developed against integrins for cancer therapy failed in clinical trials [24] possibly due to the lack of a comprehensive understanding of the major signaling pathways altered by integrin inhibitors. The heterogeneity and variability in cell signaling between different cancers is another obstacle for proposing a universal mechanism for integrin mediated tumor promotion. In the current study, we focused on describing the signaling mechanism altered by integrin deletion in pancreatic cancer. Our studies confirmed that integrin β1 promotes tumor growth in pancreatic cancer cells by increasing adhesion-mediated cell functions and pro-fibrotic signaling. Pancreatic tumor cells lacking integrin β1 protein had lower tumor forming ability in 3D collagen gels and showed reduced cell adherence and spreading on fibronectin or vitronectin. Based on TCGA data, the higher integrin β1 expression could increase integrin-dependent cell functions. In our study, the decreased integrin β1 expression inhibited pro-tumorigenic cell functions such as adhesion and spreading. The decreased expression of integrin α5 in integrin β1 KO cells corroborated the reasons for the decrease in adhesion and spread on vitronectin and fibronectin.

Multi protein macromolecular structure focal adhesions connect the intracellular actin cytoskeleton with the extracellular matrix (ECM) proteins and promote cancer progression [25]. Integrin β1 association with cytosolic proteins is necessary for the formation of nascent adhesion that further matures into focal adhesions [26,27]. Integrin’s engagement with intracellular adaptor protein kindlin-2 is an essential step in the formation and maturation of nascent adhesions [28,29]. Kindlin-2 binds to the cytoplasmic domain of integrin β1 through the conserved distal NPxY motifs and initiates the tumor promoting outside-in signaling cascade. After the association with integrins, kindlin-2 recruits another important protein molecule paxillin to nascent adhesion that is essential for the formation and functions of focal adhesions [9]. Despite the essential roles of kindlin-2 in the development of focal adhesions in epithelial cells, it is still unclear whether integrin β1 regulates kindlin-2 expression and signaling in pathological transformations. In our studies, deletion of integrin β1 from pancreatic cancer cells reduced the focal adhesion as determined by the phosphorylation status of paxillin, and downregulated kindlin-2 expression in pancreatic cancer cells. Thus, the increased expression of integrin β1 in pancreatic tumor cells could promote focal adhesion dynamics and signaling through kindlin-2 upregulation.

The higher expression of kindlin-2 has been suggested as a prognostic marker in pancreatic cancer as it associates with lower patient survival. Since the pancreatic ductal adenocarcinoma microenvironment is desmoplastic in nature, kindlin-2 associated signaling pathways that promote ECM depositions were explored. Kindlin-2 was found to promote stromal expansion by upregulation TGF-β signaling leading to Smad2/3phosphorylation [18]. Knocking down of kindlin-2 attenuated the TGF-β-stimulated Smad2/3 phosphorylation while the higher expression of kindlin-2 promoted TGF-β mediated cell signaling in fibroblasts and squamous cell carcinomas [21,30,31]. Also, a positive correlation between TGF-β and kindlin-2 has been reported in triple negative breast cancer and pancreatic ductal adenocarcinoma cells [19,32]. Our studies show that integrin β1 regulates TGF-β receptor-2 expression levels and Smad2/3 dependent intracellular cell signaling in pancreatic cancer. Phosphorylation of TGF-β receptor-2 is the major event that occurs prior to the activation of Smad transcription factors. Our data show that the expression of TGF-β receptor-2 decreased in integrin β1 KO MIA PaCa-2 cells compared to integrin β1 expressing cells. This further leads to a decrease in the phosphorylation of Smad2/3. An earlier study reported that blocking of integrin β1 inhibited the active TGF-β1 mediated MAP kinase activation [33], while our data suggests that the integrin β1 alters TGF-β receptor-2 and kindlin2 expression in pancreatic cancer and downregulates Smad2/3 phosphorylation. Integrin β1 is responsible for increased migration and cellular signaling pathways, which promote fibrosis in solid tumors. Our data suggest that by decreasing the expression of integrin β1, tumor growth is decreased. In addition, fibrosis promoting cellular signaling is reduced in KO cells. Consistent with TCGA data where lower integrin β1 expression correlated with higher survival; our data suggest that patients with lower integrin β1 expression have reduced tumor progression and increased probability of survival. Our study reveals a novel mechanism by which integrin β1 promotes tumor growth in pancreatic cancer.

## 4. Materials and Methods

### 4.1. Reagents

The MIA PaCa-2 cells used in this study were obtained from ATCC. Cell culture media and supplements, Dulbecco’s modified Eagle’s medium (DMEM) (MT10090CV; Corning, New York, USA) fetal Bovine serum (FBS) (F0926; Millipore Sigma, St. Louis, MO, USA), Horse serum (30-2041; ATCC, Manassas, VA, USA) and antibiotic antimycotic solution (30-004-Cl; Corning, New York, USA) were used. The primary antibodies used were Integrin β1 (D2E5) Rabbit mAb (9699; Cell Signaling Danvers, MA, USA), Integrin α5 antibody (4705; cell Signaling), Paxillin antibody (2542; Cell Signaling, Danvers, MA, USA), Phospho-Paxillin (Tyr118) antibody (2541; Cell Signaling), Kindlin-2 antibody (13562; Cell Signaling), TGFβR2 (E-6) (sc-17792; Santa Cruz Biotechnology, Dallas, TX, USA), Smad2/3 (A-11) (sc-393312; Santa Cruz Biotechnology, Dallas, TX, USA), Phospho-Smad2/3 (D27F4) Rabbit mAb (8828; Cell Signaling, Danvers, MA, USA).

### 4.2. Cell Culture

Cells were cultured in DMEM (MT10090CV; Corning, New York, USA) supplemented with 10% Fetal Bovine Serum (FBS) (F0926; Millipore Sigma, St. Louis, MO, USA), 2.5% Horse Serum (30-2041; ATCC) and 0.1% antibiotic antimycotic solution (30-004-Cl; Corning, New York, NY, USA). Cells were maintained in a humidified incubator containing 5% CO_2_ at 37 °C.

### 4.3. Generation of Knockout Cells

Integrin β1-KO MIA, PaCa-2 cell lines were established using Integrin β1 CRISPR/Cas9 knock out Plasmids (sc-421171-NIC-2: Santa Cruz Biotechnology, Dallas, TX, USA). Cells were transfected using Invitrogen™ Lipofectamine™ 3000 Transfection Reagent (L3000015; Fisher Scientific, Waltham, MA, USA) in DMEM media. Cells were incubated with CRISPR/Cas9 plasmid for 5 h at 37 °C in a CO_2_ incubator. Following incubation, normal growth media were added to culture the cells for 48 h. Cells were selected with media containing 1 µg/mL puromycin (97064-280; VWR, Radnor, PA, USA). Individual clones were selected with cloning cylinder. Selected clones were cultured and immunoblotting was performed to compare the expression of integrin β1 with WT.

### 4.4. Immunoblotting

Cells were cultured to attain 70–80% confluence in regular media. Cells were washed twice with PBS and lysed using cell lysis buffer (9803S; Bio-rad, Hercules, CA, USA) containing protease and phosphatase inhibitor cocktail (1861281; Thermo Fisher Scientific, Waltham, MA, USA). Whole cell lysates were mixed for 30 min at 4 °C, centrifuged at 15000 rpm for 15 min at 4 °C, and supernatant was collected. Protein concentrations were determined using the bicinchoninic acid (BCA) Protein Assay Kit (23225; Thermo Fisher Scientific, Waltham, MA, USA). An amount of 30 μg of total protein samples was mixed with 2× SDS sample buffer and loaded into Bio-Rad 4–20% precast polyacrylamide gel, 8.6 × 6.7 cm (4561093; Bio-Rad, Hercules, CA, USA). For Western blotting, protein samples were transferred to 0.2 µm polyvinylidene difluoride (PVDF) membranes (88520; Thermo Fisher Scientific, Waltham, MA, USA). Membranes were blocked with 5% fat free milk (SC-2325; Santa Cruz Biotechnology, Dallas, TX, USA) in Tris buffer saline (TBS) with 0.1% Tween-20 (sc-29113; Santa Cruz Biotechnology, Dallas, TX, USA) (TBST) for 1 h, washed three times with TBST and incubated overnight at 4 °C with the primary antibodies. Antibodies were diluted in 5% Bovine Serum Albumin (BSA) (BP1600; Fisher Scientific, Waltham, MA, USA) in Tris buffer saline (TBS) with 0.1% Tween-20 (TBST) following optimized concentration. Immunoblotting analysis was visualized using the enhanced chemiluminescence (ECL) or ECL plus in the Western blotting detection system. GAPDH expression was determined for loading control. The expression level of proteins was quantified using imageJ software (V:1.53C NIH, Bethesda, MD, USA).

### 4.5. RNA Isolation and RT PCR

Total RNA was isolated from cells using RiboZol RNA Extraction Reagent (N580; VWR, Radnor, PA, USA) following the manufacturer’s protocol. cDNA synthesis was performed with 1 µg RNA using the OneScript cDNA synthesis kit (G-234; abmgoods, Applied Biological Materials Inc., Richmond, BC V6V 2J5, Canada). Real-time PCR (RT-PCR) was performed in triplicate for each sample with Brightgreen 2X qPCR MasterMix-No dye (MasterS; abmgoods, Applied Biological Materials Inc., Richmond, BC V6V 2J5, Canada). Each reaction was normalized against 18S. mRNA expression was interpreted as fold change compared between cells. Primer sequences are as shown in Table 1.

### 4.6. Immunocytochemistry for Focal Adhesion Assay

Immunofluorescence images were recorded with an Zeiss LSM 900 confocal microscope (Carl Zeiss Microscopy, LLC, Thornwood, NY, USA). Cells were grown to 60–70% confluence on glass coverslips in DMEM (MT10090CV; Corning) medium containing 10% fetal bovine serum (FBS), 2.5% Horse serum 0.1% antibiotics. Cells were rinsed with PBS containing 1.0 mM CaCl_2_ and 0.5 mM MgCl_2_ and fixed by 4% paraformaldehyde for 15 min. Cells were washed and blocked with blocking buffer for 1 h at room temperature. The primary antibodies for paxillin and phospho-paxillin were used at 1:200 in TBST and allowed to bind with proteins at 4 °C overnight. Alexa Flour 555 tagged secondary 1:1000 was incubated at room temperature for 2 h. To stain cytoskeletal F-actin, the cells were incubated with ActinGreen™ 488 Ready Probes™ Reagent (AlexaFluor™ 488 phalloidin) (R37110; Thermo Fisher Scientific, Waltham, MA, USA) in antibody buffer for 30 min. Cells were mounted with ProLong™ Gold Antifade Mountant with DAPI (for nucleus) (P36931; Thermo Fisher Scientific, Waltham, MA, USA).

### 4.7. Cell Spreading Assay

Spreading ability of cells on different ECM proteins was measured using cell spreading assay. Glass coverslips were coated with the desired ECM overnight at 4 °C, blocked with 0.5% heat denatured BSA (BP1600; Fisher Scientific, Waltham, MA, USA) for 1 h. Five thousand cells were plated and left for 4 h at 37 °C. The cells were fixed with 4% paraformaldehyde for 15 min at room temperature, blocked and stained with ActinGreen™ 488 Ready Probes™ Reagent. ProLong™ Gold Antifade media with DAPI (for nucleus) (P36931; Thermo Fisher Scientific, Waltham, MA, USA) was used to mount the cells. Images were obtained with a confocal microscope (Carl Zeiss Microscopy, LLC, Thornwood, NY, USA). The cell area was assessed with ImageJ software (Version: 1.53c, National Institutes of Health, Bethesda, MD, USA). The results are expressed as average area ± SEM.

### 4.8. Cell Adhesion Assay

The ability of cells to adhere to selected ECM proteins was measured using cell adhesion assay. The 96-well plates were coated with the desired ECM at the indicated concentrations at 4 °C overnight. Before the assay, plates were incubated with 0.5% heat denatured BSA in PBS for 60 min to block non-specific adhesion. FBS containing wells were used as positive controls to determine the total cell loaded and normalize. 5 × 10^5^ cells seeded in serum-free DMEM media and incubated for 60 min at 37 °C. Non-adherent cells were removed by washing the wells with PBS. Cells were fixed with 4% paraformaldehyde and stained with 1% Janus green for 5 min at room temperature. Cells were washed and 100 µL of 0.5M HCl was added to lyse the cells in each well. The number of cells adhered was measured at 595 nm. Cells bound to FBS coated wells (positive control) were used to indicate 100% adhesion and cells bound to 0.5% heat denatured BSA coated wells (negative control) were used to evaluate background binding. The percentage of cells adhered to the plates was expressed as percentage cell adhesion. Average percent cell adhesion was expressed as mean± SEM.

### 4.9. 3D Matrix Cell Proliferation and Colony Growth

Cells were grown in a matrix containing matrigel and collagen for 7 days under cell culture conditions. The 3D matrix was harvested and fixed with 4% paraformaldehyde at room temperature and stained for actin as described in the case of cell spreading assay. Images were obtained using LSM 900 confocal microscope in z-stack. Tumor 3D projection was obtained using ZEN 3.1 (blue edition) software. Tumor volume was measured using ImageJ software plug-in described below.

Volume determination of in vitro 3D cell colonies:

*Loading Image Stacks*: Three-dimensional confocal imaging results in a collection of images as planes incrementing along the Z axis, known as an “image stack” (also known as a “z-stack”). We loaded our Z-Stack (Zeiss .czi files) into ImageJ by using the *File > Import > Bioformats* command. Opened the z-stack in ImajeJ by going to *File > Open Samples > Confocal Series* menu option.

*Threshold of the Image*: To select the threshold of the image, follow Image > Adjust > Threshold (or hit Ctrl + Shift + T) to bring up the Threshold dialog. We selected automatic threshold and Otsu method from the automatic threshold dropdown box. The Otsu threshold method is very useful for separating foreground objects from background in microscopy.

*Set Measurements*: Next, Select *Analyze > Set Measurements* menu option to get the set measurements dialogue and selected area stack position, Mean gray value, Stack position, and Limit to Threshold.

*Macro loop*: Then, we used a macro to measure each image in the stack and calculate the volume. The macro will loop through each image in the stack and take the measurement of each slice individually. Finally, the macro will loop through and sum the area measurements, multiply this sum by the depth of each slice, and finally display the result. The macro loop was created by selecting *Plugin > New > Macro* menu item in ImageJ and putting the below mentioned code into the macro window.

- // ImageJ Macro Code
- // Measure Volume of Thresholded Pixels in an Image Stack
- //
- //
- macro "Measure Stack" {
- run("Clear Results"); // First, clear the results table
- // loop through each slice in the stack. Start at n=1 (the first slice),
- // keep going while n <= nSlices (nSlices is the total number of slices in the stack)
- // and increment n by one after each loop (n++)
- for (n=1; n<=nSlices; n++) {
- setSlice(n); // set the stack’s current slice to n
- run("Measure"); // Run the "Measure" function in ImageJ
- }
- // Create a variable that we will use to store the area measured in each slice
- totalArea = 0;
- // Loop through each result from 0 (the first result on the table) to nResult (the total number of results on the table)
- for (n=0; n < nResults; n++)
- {
- totalArea += getResult("Area",n); // Add the area of the current result to the total
- }
- // Get the calibration information from ImageJ and store into width, height, depth, and unit variables.
- // We will only be using depth and unit
- getVoxelSize (width, height, depth, unit);
- // Calculate the volume by multiplying the sum of area of each slice by the depth
- volume = totalArea*depth;
- // Print the result of the volume calculation to the log
- print(volume + " " + unit + "^3");
- }

### 4.10. Statistical Analysis

Data is represented in mean ± SEM. To determine the statistical significance, analysis of variance (ANOVA) followed by Bonferroni’s post-hoc test. At least 3 independent experiments were carried out. Differences were considered to be statistically significant at the *p* < 0.05 levels.

## Figures and Tables

**Table 1:** Primers used for the gene expression using qRTPCR analysis.

Gene	Forward Primer Sequence	Reverse Primer Sequence
ITGα5	5′-TGCAGTGTGAGGCTGTGTACA-3′	5′-GTGGCCACCTGACGCTCT-3′
Kindlin 2	5′-GTCCCCGCTATCTAAAAAAGT-3′	5′-GATGGGCCTCCAAGATTCT-3′
TGFβR2	5′-ATGACATCTCGCTGTAATGC-3′	5′-GGATGCCCTGGTGGTTGA-3′
18S	5′-GTAACCCGTTGAACCCCATT-3′	5′-CCATCCAATCGGTAGTAGCG-3′

## Supplementary Materials

The following are available online at https://www.mdpi.com/article/10.3390/ijms221910599/s1.
