# Supplementary material for: Integrin β1 Promotes Pancreatic Tumor Growth by Upregulating Kindlin-2 and TGF-β Receptor-2
Source: Int J Mol Sci. 2021 Sep 30;22(19):10599. doi: 10.3390/ijms221910599 (PMC8508632; doi:10.3390/ijms221910599)
Supplement: Supplementary file 1 [file ijms-22-10599-s001.zip › ijms-1385601-supplementary.pdf]

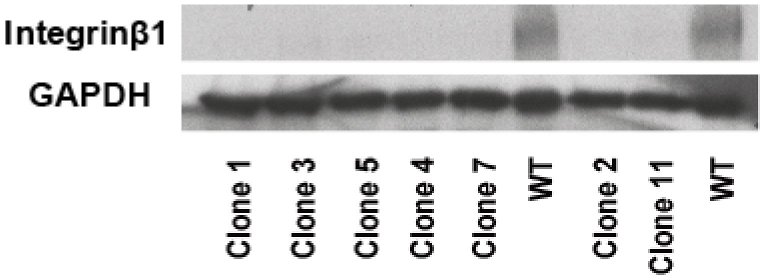

Supplementary Figure S1: Selection of β1 knockout MIA PaCa-2 clones. Clones were selected after shRNA treatment by puromycin selection. Selected clones were screened for the expression of integrin β1 by immunoblotting. WT MIA PaCa-2 samples were used to compare between the levels of expression.
